# Employment and disability status in patients with functional (psychogenic nonepileptic) seizures

**DOI:** 10.1002/brb3.2016

**Published:** 2020-12-29

**Authors:** Ali A. Asadi‐Pooya, Mehdi Bazrafshan

**Affiliations:** ^1^ Epilepsy Research Center Shiraz University of Medical Sciences Shiraz Iran; ^2^ Jefferson Comprehensive Epilepsy Center Department of Neurology Thomas Jefferson University Philadelphia PA USA

**Keywords:** disability, employment, functional, psychogenic, seizure

## Abstract

**Purpose:**

We investigated the rate of employment in patients with functional seizures (FS) in a follow‐up study. We also investigated the rate of receiving disability benefits in these patients. Finally, we investigated factors that are potentially associated with their employment status.

**Methods:**

In this long‐term study, all patients with FS, who were diagnosed at Shiraz Comprehensive Epilepsy Center, Iran, from 2008 to 2018, were investigated. In a phone call interview to the patients in February 2020, we tried to obtain the following information: seizure outcome, employment status, receiving disability benefits, and their current drug regimen, if any. The first call was made in the evening and after working hours. In case of no response, we made two more attempts in the following weeks to contact the patients during different time periods of the day.

**Results:**

Eighty‐ four patients participated. Thirty‐one patients (37%) were employed, and 53 people (63%) were not; at the first visit, the rate of employment was 23%. Female sex (Odds Ratio [OR]: 12.18; 95% Confidence Interval [CI]: 3.51–42.18; *p* = .0001), taking psychiatric drugs (OR: 4.93; 95% CI: 1.17–20.73; *p* = .02), and being employed previously (OR: 0.19; 95% CI: 0.04–0.77; *p* = .02) were independently significantly associated with the current employment status. Three patients (4%) reported receiving disability social benefits, two women and one man.

**Conclusion:**

This study highlights that unemployment is a serious issue in patients with FS and psychiatric comorbidities play a significant role in the employment status in these patients.

## Graphical abstract

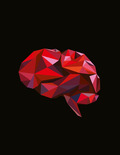

## 1 INTRODUCTION

Functional seizures (FS) or psychogenic nonepileptic seizures (PNES) are characterized by paroxysmal and self‐limited events that semiologically may resemble epileptic seizures, but without ictal epileptiform discharges; they are considered as psychological problems (Asadi‐Pooya et al., 2020). While there is no universally accepted terminology for this common condition, “functional seizures” meets more of the criteria proposed for an acceptable label than other popular terms in the field (e.g., PNES). Hence, we have adopted “functional seizures” terminology in the current paper (Asadi‐Pooya, Brigo, et al., 2020).

Many patients with FS experience loss of responsiveness with their seizures (Asadi‐Pooya & Bahrami, 2019). Hence, it is plausible to assume that people with FS might be at increased risk of experiencing job‐related difficulties (Nazeri et al., 2020). A few previous studies (all from the Western and developed countries) have shown that patients with FS have low rates of employment and may be receiving some form of social financial support (Jennum et al., 2019; McKenzie et al., 2016; Walczak et al., 1995). In a study of 120 patients with FS from the UK, only 30% were employed 5–10 years postdiagnosis (McKenzie et al., 2016).

In the current endeavor, we investigated the rate of employment in patients with FS living in a developing nation from the Middle‐East, in a follow‐up study. We also investigated the rate of receiving disability benefits in these patients. We hypothesized that unemployment is common in these patients and some of these patients are receiving disability benefits. Also, we investigated factors that are potentially associated with the employment status in patients with FS living in a developing nation. This data may add to the literature and improve our understanding of the social status of patients with FS cross‐culturally.

## 2 MATERIALS AND METHODS

In this long‐term follow‐up study, we investigated all adult patients with FS admitted to the epilepsy monitoring unit at Shiraz Comprehensive Epilepsy Center from December 2008 through September 2018. All included patients were 20 years of age or older at the time of their first visit. Patients had a confirmed diagnosis of FS, determined by clinical assessment and video‐EEG monitoring with ictal recording of their seizures. We excluded patients with comorbid epilepsy, abnormal electroencephalography (EEG), or insufficient data. We extracted all the relevant clinical and demographic data at the time of diagnosis from our database (age, sex, seizure semiology [e.g., aura, loss of responsiveness with functional seizures, generalized motor seizures], medical comorbidities [non‐neurological and nonpsychiatric medical comorbidities such as diabetes, heart disease, etc.], family history of seizures, employment, marriage, taking antiseizure medications, and taking psychiatric medications). In a phone call interview to the patients in February 2020 (i.e., at least 18 months after their first visit), we tried to obtain the following information if patients agreed to participate and answer the questions (consecutively, we asked the following questions): seizure outcome (seizure‐free during the past 12 months or not), employment status, receiving disability benefits, and their current drug regimen, if any. The first call was made in the evening and after working hours (i.e., 6–9 p.m.). In case of no response, we made two more attempts in the following weeks to contact the patients during different time periods of the day (i.e., 11 a.m.–13 pm and 4–6 p.m.).

We first studied factors associated with their current employment status using Pearson chi‐square and Fisher's exact tests. Variables that were significant in univariate tests were assessed in a logistic regression model. Odds ratio and 95% confidence interval (CI) were calculated. *p* values < .05 were considered significant. The study design was reviewed and approved by Shiraz University of Medical Sciences Institutional Review Board and ethical committee. Oral informed consent of the participants was obtained after the nature of the procedures had been fully explained.

## 3 RESULTS

During the study period, 198 patients with FS‐only had available phone numbers and other inclusion criteria and were approached. One hundred and eight people did not answer our call, three persons declined to participate, and three people were dead. Eighty‐four patients participated in this study. They included 52 female and 32 male patients. The mean age of the participants (±standard deviation) at the time of diagnosis was 30 (±9) years (range: 20–53 years). In the follow‐up call, 40 patients (48%) were seizure‐free and 44 patients (52%) were still suffering from functional seizures. Thirty‐one patients (37%) were employed, and 53 people (63%) were not; at the first visit, the rate of employment was 23% (19 out of 84 patients). Table 1 shows the variables in association with the current employment status in univariate analyses. Seizure characteristics and seizure control status were not associated with current employment status, but sex, primary employment status, and taking psychiatric drugs were significantly associated with current employment status in univariate analyses. We then analyzed the association between the current employment status and variables with a *p* < .05 in a binary logistic regression model. The model that was generated by regression analysis was significant (*p* = .0001) and could predict the current employment status in 83% of the patients. Within this model, female sex (OR: 12.18; 95% CI: 3.51–42.18; *p* = .0001), taking psychiatric drugs (OR: 4.93; 95% CI: 1.17–20.73; *p* = .02), and being employed previously (OR: 0.19; 95% CI: 0.04–0.77; *p* = .02) were independently significantly associated with the current unemployment; women and patients currently taking psychiatric drugs were more often unemployed and those, who were employed at the time of the diagnosis, were more often employed in the follow‐up.

**TABLE 1:** Variables in association with employment in univariate analyses

	Employed (*N* = 31)	Unemployed (*N* = 53)	*p* value
Sex (female: male)	8:23	44:9	.0001
Age at diagnosis (mean ± standard deviation) years	28.9 ± 7.8	30.9 ± 10.3	.3
Aura with functional seizures	22 (71%)	40 (75%)	.7
Loss of responsiveness with seizures	25 (81%)	43 (81%)	1.0
Generalized motor seizures	29 (94%)	46 (87%)	.4
Medical comorbidity	6 (19%)	15 (28%)	.4
Family history of seizures	8 (26%)	16 (30%)	.8
Seizure‐free in the past 12 months	17 (55%)	23 (43%)	.3
Previous employment	15 (48%)	4 (8%)	.0001
Married at the time of diagnosis	17 (55%)	35 (66%)	.3
Married at the time of follow‐up call	24 (77%)	41 (77%)	.5
Taking antiseizure medications	9 (29%)	12 (23%)	.6
Taking psychiatric medications	5 (16%)	24 (45%)	.009
Receiving psychological care	12 (39%)	29 (55%)	.1

Among the whole cohort of patients with FS in the follow‐up, three patients (4%) reported receiving disability social benefits, two women and one man. These three people were unemployed at the time of their first visit, and all were not seizure‐free in their follow‐up.

## 4 DISCUSSION

Functional seizures affect many aspects of a person's life. This condition may have substantial socioeconomic consequences for patients, their partners, and the society (Jennum et al., 2019). In this study, we observed that the majority of patients with FS were unemployed and this situation did not improve significantly with the passage of time (from 23% at the first visit to 37% at the last follow‐up call). In a previous study of patients with FS from the UK, only 30% were employed 5–10 years postdiagnosis (McKenzie et al., 2016). This is consistent with our finding. Furthermore, we observed that achieving seizure freedom does not have association with the employment status in the long‐term, but taking psychiatric drugs (an indirect evidence of having a psychiatric comorbidity) was significantly associated with the current employment status. In the UK study (McKenzie et al., 2016), unemployment was predicted by contact with psychiatric services at 5–10 years (OR: 0.16, CI: 0.05–0.58, *p* = .005). This observation corroborates our finding of the significance of psychiatric comorbidities in association with the employment status. These data suggest that in patients with FS, psychiatric comorbidities play a significant role in the employment status and targeting these comorbidities in the treatment plan of patients may potentially help them achieve a lot socially. This proposition should be studied in the future. However, it is advised to look carefully for mood disturbances, personality disorders, and psychic trauma in people with FS and consider cognitive‐behavioral therapy and pharmacological treatment to manage comorbid psychiatric conditions, namely anxiety and depression. Functional seizure management should be multidisciplinary (Gasparini et al., 2019).

In the current study, female sex had a significant association with the employment status. This probably has more socio‐cultural reasons than biological underpinnings. In Iran, while women are legally permitted to work in almost any job they want (as men are), men are culturally responsible to provide for the family. In addition, there exists social discrimination against women in finding a job (in contradiction with the above legal status). In other words, laws generally do not discriminate against women in the job market, but policies do! Labor force participation rates in the fiscal 2016–2017 were 64% of men and 15% of women in Iran (https://financialtribune.com/articles/economy‐business‐and‐markets/78671/iran‐s‐women‐labor‐force‐participation‐lowest‐worldwide/. Accessed on March 26, 2020); the employment rates were 15% in women and 72% in men with functional seizures in our study. We also observed that the employment status at the time of the diagnosis had a significant association with the employment status in the follow‐up. This finding is pretty much expected; many of those (15 out of 19; 79%), who had a job at the time of the first visit, managed to remain employed in the long term. However, only a minority (16 out of 65; 25%) of the unemployed patients (at the first visit) managed to get employed with the passage of time. The potential socio‐biological reasons for this observation should be explored in future studies.

Finally, we observed that only a small minority of patients with FS (4%) were receiving social support and disability benefits, despite the high rate of unemployment in this study. In a study by the international league against epilepsy (ILAE) PNES Task Force, no respondent from low‐income countries stated that their patients could receive state disability benefits for FS, compared to 23% in the middle‐income and 50% in high‐income countries (Hingray et al., 2018). In a previous study, we observed that 75% of the physicians in our area believed that patients with FS and specific jobs or professions (e.g., pilots, bus drivers, and firefighters) should be qualified for disability benefits, as long as they have active FS (Asadi‐Pooya et al., 2020). However, laws and regulations in Iran do not consider patients with FS eligible to receive social support and disability benefits, while patients with drug‐resistant epileptic seizures could be qualified to receive disability benefits in Iran! Ironically, functional seizures are associated with significantly higher health‐related and other costs, and lower levels of employment and income than those in patients with epilepsy (Duncan et al., 2014; Jennum et al., 2019). Furthermore, a major risk factor for early disability pension in patients with epilepsy is psychiatric comorbidity, (Specht et al., 2015) and more patients with FS suffer from other psychiatric comorbidities (e.g., depression and anxiety) than those with epilepsy (Walsh et al., 2018). This paradox is likely related to the economic status, public politics, and social and cultural issues, particularly in low‐income countries. Experts in the field and patient support organizations should negotiate with the authorities to improve the current laws and regulations on the issue of social support eligibility in patients with FS all around the world.

This study has some limitations. The sample size was not large. Furthermore, self‐report nature of the questionnaire might have influenced the results. In addition, we did not include all the possible variables that might be related to the employment status (e.g., living status). However, this study highlights that unemployment is a serious issue in patients with FS and psychiatric comorbidities play a significant role in the employment status in these patients.

## CONFLICT OF INTEREST

Ali A. Asadi‐Pooya, M.D.: Honoraria from Cobel Daruo, RaymandRad and Tekaje; Royalty: Oxford University Press (Book publication). Mehdi Bazrafshan: none. None of the authors listed on the manuscript are employed by a government agency. All are academicians. None of the authors are submitting this manuscript as an official representative or on behalf of the government.

## AUTHOR CONTRIBUTIONS

Ali A. Asadi‐Pooya, M.D. involved in design and conceptualized the study, analyzed the data, drafted and revised the manuscript. Mehdi Bazrafshan involved in data collection and revised the manuscript.

### Peer Review

The peer review history for this article is available at https://publons.com/publon/10.1002/brb3.2016.

## Data Availability

The data use in this study is confidential and will not be shared.
